# Gubenzhike Recipe Ameliorates Respiratory Mucosal Immunity in Mice with Chronic Obstructive Pulmonary Disease through Upregulation of the *γδ*T Lymphocytes and KGF Levels

**DOI:** 10.1155/2020/3056797

**Published:** 2020-03-24

**Authors:** Yue-qi Wang, Qiang Liao, Shi-huan Tang, Zhe Cai, Hong-chun Zhang

**Affiliations:** ^1^Beijing University of Chinese Medicine, Beijing 100029, China; ^2^China-Japan Friendship Hospital, Beijing 100029, China

## Abstract

**Background:**

Gubenzhike recipe, a traditional Chinese herbal compound, was assumed to have a possible beneficial effect on COPD. This study was designed to elucidate the mechanism from the perspective of respiratory mucosal immunity.

**Methods:**

COPD model was induced by exposure to cigarette smoke and LPS instillation in mice for 12 weeks. Animals were administered solution of Gubenzhike recipe by intragastric gavage daily for 4 weeks. After that, mice were sacrificed for lung function test and histological examination of lung tissues. The levels of IL-6 and IL-13 in serum, bronchoalveolar lavage fluid (BALF), and intestinal mucus were measured by ELISA. The KGF and KGFR in lung tissue were analysed by immunohistochemical staining, ELISA, and western blotting, and the mRNA expressions were assessed by PCR. *γδ*T lymphocytes in the lungs were isolated and analysed by immunohistochemical staining and flow cytometry.

**Results:**

Gubenzhike recipe improved the structure of airway and damage of lung tissue and also the respiratory status and lung function, reduced the content of IL-6 in serum and BALF and IL-13 in BALF and intestinal mucus, increased the proportion of *γδ*T cells in lung tissue, and promoted the secretion of KGF and KGFR (*P* < 0.05).

**Conclusion:**

We for the first time demonstrated an experimental procedure for the isolation of *γδ*T lymphocytes from lung tissue. This study suggested that Gubenzhike recipe could enhance the respiratory mucosal immunity which provided experimental evidence for its effects of reinforcing “wei qi” by means of strengthening vital qi, tonifying spleen and kidney, relieving cough, and reducing phlegm in TCM.

## 1. Introduction

Chronic obstructive pulmonary disease (COPD), characterized by persistent respiratory symptoms and airflow limitation caused by airway and/or alveolar abnormalities, is a preventable and treatable respiratory system disease, also a common cause of death worldwide associated with a significant socioeconomic burden. COPD progresses slowly over decades with prolonged functional decline in the respiratory system. Ultimately, patients die from respiratory failure or preceding complications, such as cardiovascular disease and lung cancer [1].

The pathological characteristics of COPD are visible in the airways, pulmonary vasculature, and lung parenchyma, including chronic inflammation with increasing nonspecific inflammatory cells in different parts of the lung, and the structural changes due to repeated injury repair. These changes aggravate with the progression of the disease and continue to exist even after smoking cessation. Increasing circulating cytokines, chemokines, and acute-phase proteins are evidence of systemic inflammation and may cause systemic damage and complications in patients with COPD. A large sample study showed that the risk of cardiovascular disease, pneumonia, diabetes, and lung cancer increased by 2–4 folds with a systemic inflammation measured by six inflammatory markers (CRP, IL-6, CXCL8, fibrinogen, TNF-*α*, and leukocytes). It was shown that 70% of patients with COPD had a component of systemic inflammation, 16% had persistent inflammation, and these patients had a higher frequency of mortality and acute exacerbations [2]. In particular, mononuclear macrophages and vascular endothelial cells can release IL-6, IL-13, and other inflammatory factors after stimulated in patients with COPD. Inflammatory reactions thereby take place locally in the airways and lungs and systemically with increased levels of inflammatory factors in circulation. Studies have shown that the increase of IL-6 may play a vital role in the continuous inflammation of airways, hypersecretion of mucus, progressive destruction of lung tissues, and other diseases. When binding to functional receptors, IL-13 can aggrandize IgE, eosinophilic inflammation, airway hyperresponsiveness, bronchial goblet cell secretion, expression of various chemokines and adhesion molecules, and airway epithelial fibrosis. IL-13 can affect the secretion of secretory cells and the function of airway cilia by increasing the proportion of secretory cells and causing differentiation changes of airway cilia cells and consequently makes the airway damaged and blocked [3].

A layer of mucosal epithelial cells covers the surface of the respiratory tract, forming a physical and immune barrier to prevent the invasion of nonautogenous harmful substances. Mucous membrane needs to distinguish not only beneficial and harmful substances, but also resident and pathogenic bacteria. The mucosal immune system (MIS) is needed in order to control the mechanism that beneficial substances or resident bacteria need to be actively used, while harmful substances or bacteria need to be selectively eliminated [4].

To clear pathogens in the respiratory tracts, there are immune responses taking place in a variety of lymphoid tissues which promote local immune responses in different parts of upper and lower respiratory tracts. Lower airways of both humans and mouse lack the developmentally formed submucosal lymphoid tissues, but have bronchus-associated lymphoid tissue (BALT) that deals with inflammation or infection. BALT distributes among the lung and has a wide diversity of structures, ranging from fully differentiated lymphoid tissue to isolated B-cell follicles without adjacent T-cell areas, to small clusters of B cells with little apparent organization. Exposure to cigarette smoke is an important risk factor for COPD, and the frequency of BALT was higher in smokers than in nonsmokers, and higher in smokers with significant airway obstruction than in smokers without that. Exposure to cigarette smoke increased the BALT area and its contact with epithelial cells in rats by nearly four times. The most significant increase in BALT was showed in patients classified with GOLD stage 3 and stage 4 disease. The change of FEV1 (forced expiratory volume in one second) correlates strongly with the percentage of airways with lymph follicles, suggesting that BALT might play an important role in COPD [5]. In patients with COPD, the numbers of T lymphocytes in the lung parenchyma, peripulmonary tissue, and central airway were all increased, and the number of T cells was related to the number of alveolar destruction and the severity of airway obstruction. In addition, the only noteworthy difference in inflammatory cell infiltration between asymptomatic smokers and smokers with COPD was an increase in T cells [2]. T cells are the main lymphocytes, and *γδ*T cells are one of the primary T cells to develop in the thymus. The majority of T cells in humans and mouse are *αβ*T cells. *γδ*T cells make up only a small percentage (1–5%) of circulating T cells in the blood and secondary lymphoid organs, but the proportion is much higher (10–100%) in epithelial tissues such as the skin, digestive tract, and reproductive tract. The large number of cells presenting at specific localization suggests that these cells are associated with epithelial or mucosal immunity. *γδ*T cells express tissue-specific T-cell receptors, which connect innate and adaptive immunity and play a protective role in immune surveillance [6, 7]. *γδ*T cells can synthesize and secrete KGF, regulate IL-17, IL-22, and other cytokines, recruit neutrophils and platelets, increase vascular endothelial growth factor (VEGF) which is needed for nerve regeneration, participate in tissue repair, promote epithelial healing, and regulate epithelial homeostasis [6].

In the preliminary clinical study, we observed that the clinical symptoms of patients with chronic bronchitis were significantly improved after taking the Gubenzhike recipe. And previous animal experiments have shown that Gubenzhike recipe can relieve the restriction of respiratory tracts, adjust the biological activity of *γδ*T cells, reduce the IL-17, and increase KGF in lung in mouse models. Local inflammation and process of damage-repair in the airway were reduced in mice treated by Gubenzhike recipe. In conclusion, its mechanisms of action on mice with COPD are promoting to repair the damage of inflammation in lung tissue, enhancing the respiratory mucosal immunity, and improving the integrity of airway [8–11]. The main pathogenesis of COPD is inflammatory and repeated damage-repair, while *γδ*T cells have functions of restoring inflammatory injury, killing inflammatory cells, and promoting wound repair. However, few studies focus on the effect of Chinese traditional medicine on the *γδ*T cells. In this study, we proposed the regulatory effect of Gubenzhike recipe on *γδ*T cells of respiratory mucosal immunity is part of the mechanisms of treating COPD.

## 2. Materials and Methods

### 2.1. Animals

A total of 125 healthy SPF grade ICR female mice, weighing 18∼20 g and aged 6∼8 weeks, were provided by Beijing Huafukang Biotechnology Co., Ltd (Animal production license number: SCXK (Beijing) 2014-0004). This experiment complied with the recommendations of the Guidelines for the Care and Use of Laboratory Animals of the Ministry of Science and Technology of China. All the protocols were reviewed and approved by the Institutional Animal Experimental Ethics Committee of China-Japan Friendship Hospital. The animals were raised at the experimental animal platform of China-Japan Friendship Hospital (license no. SYXK (Beijing) 2016-0043). All mice were accommodated with free access to food and water. After adaptive feeding for 1 week, they were randomly divided into 5 groups: blank control group, model group, high-dose Gubenzhike recipe group, medium-dose Gubenzhike recipe group, and low-dose Gubenzhike recipe group, with 25 mice in each group.

### 2.2. Gubenzhike Recipe

Astragalus, Epimedium, Rhizoma Atractylodes, Radix Stemonae, Radix Scutellariae, Radix Paeoniae Rubra, and Radix Saposhnikoviae were mixed at a ratio of 4 : 3 : 2 : 3 :3 : 2 : 1. Dry extract containing 3.6 g raw herb per milliliter was made in the Department of Pharmaceutical Preparation of China-Japan Friendship Hospital. Then, we calculated the dose depending upon the per kg body mass dose conversion coefficient table between animals and adults. The high-dose solution, medium-dose solution, and low-dose solution were administrated at 3.5 g raw herb/kg, 1.8 g raw herb/kg, and 0.58 g raw herb/kg bodyweight of mouse separately.

### 2.3. Animal Model

Mice were exposed to successive periods of cigarette smoke (CS) for 7 days/week for 12 weeks (except the days using LPS). The stability of the model was verified by morphology and functional analysis of several animal experiments early. The cigarettes were purchased from Shanghai Tobacco Co., Ltd (12 mg tar oil in each cigarette, 0.9 mg nicotine, and 14 mg carbon monoxide in flue gas). The mice were put in a glass box (70 cm × 50 cm × 30 cm), and then 10 cigarettes were lighted in the box and burnt for 10 min till burn-out. There was 5 min interval to open the box for air and then the above steps were repeated once. The LPS (1.5 mg/kg) (L2880, Sigma-Aldrich, St. Louis, MO, USA) in distilled water was implemented by instillation through the nasal cavity to respiratory tract on day 1, day 29, and day 57. Mice's weight was measured every 4 weeks and their health status was monitored. After the model establishment, an equal volume (0.2 ml/day/animal) of distilled water or solution of dry extract of Gubenzhike recipe was administered separately by oral gavage to the blank control group, model group, and Gubenzhike recipe groups once a day for 4 weeks [9, 10, 12, 13].

### 2.4. Lung Function Test

2% pentobarbital solution was intraperitoneally injected to anesthetize mice (0.1 ml/10 g). Mouse was fixed in the supine position on an operating table. The neck skin was cut and the trachea was intubated following blunt separation. The tube was fixed with a cotton thread and connected to ventilator. Lung function was measured by a small-animal ventilator (flexiVent; Scireq, Montreal, QC, Canada). After conventional mechanical ventilation, single-compartment model (snap-150) and constant phase model (prime-8) were run to measure lung function. The parameters recorded are shown in Table 1.

### 2.5. Collection of BALF, Serum, Intestinal Mucus, and Lung Tissue Samples

After the pulmonary function test, a 1 ml syringe was connected to endotracheal intubation for irrigation. 0.8 mL sterile PBS (SH30256.01, Hyclone, South Logan, UT, USA) was slowly injected each time and the operation was repeated 3 times. The lavage solution was collected and centrifuged at 3000 rpm for 15 min. The supernatant was taken and stored at −80°C for testing. 0.8–1 ml blood was taken from the heart and then subjected to 37°C water bath for 15 min, refrigerated at 4°C for 15 min, and centrifuged at 3000 rpm for 15 min. The upper serum was collected and stored for use at −80°C. The upper left lung lobe from nonlavaged lungs was taken and fixed in 10% formaldehyde solution for at least 24 h. After dehydration, the lung lobe was embedded in paraffin and sliced. The right lung was excised and kept in liquid nitrogen before tests. The abdominal cavity was opened to take the entire small intestine between pylorus and cecum. The intestinal cavity was rinsed with 10 ml of sterile PBS. The rinse solution was centrifuged at 3000 rpm for 15 min, and the supernatant was preserved at −80°C for testing.

### 2.6. Morphological Observation of Lung and Expression Detection of *αβ*TCR, *γδ*TCR, KGF, and KGFR

Hematoxylin-eosin (HE) staining was performed on the sections after being dewaxed in xylene and rehydrated through descending series of ethanol concentrations. Changes of morphology and submucosal lymphocytes were observed under an upright light microscope (Olympus, Tokyo, Japan). The sections were incubated at 0.3% H_2_O_2_ (ZLI-9311D, Zhongshan Golden Bridge Biotechnology Co., Ltd., Beijing, China) for 15 min to block endogenous peroxidase activity. After washing three times with PBS for 5 min each time, the sections were incubated with antibodies of *αβ*TCR (1 : 100 dilution, ab25201, Abcam, Cambridge, MA, USA), *γδ*TCR (1∶100 dilution, ab25209, Abcam, Cambridge, MA, USA), KGF (1∶800 dilution, orb10652, Biorbyt, Cambridge, UK), and KGFR (1 ∶ 300 dilution, ab10648, Abcam, Cambridge, MA, USA) overnight at 4°C. On the second day, the sections were incubated with the goat anti-rabbit IgG H&L (HRP) antibody (zb2305, Zhongshan Golden Bridge Biotechnology Co., Ltd., Beijing, China) at room temperature for 1 h in the moisturizing box. Reaction was then visualized using diaminobenzidine (DAB) (Zhongshan Golden Bridge Biotechnology Co., Ltd., Beijing, China), and color development was controlled under a microscope. Between each of the above steps, sections were washed with PBS for 10 minutes. Finally, sections were counterstained with hematoxylin. Positive cells were counted in three random fields at 400x magnification in each section.

### 2.7. Western Blot and ELISA

The lungs were collected and crushed in a sterile mortar filled with liquid nitrogen. 50 mg lung tissue was weighed and 600 *μ*l NP40 lysate (P0013F, Beyotime Biotechnology, Shanghai, China) was added. After crushing the tissue for 30 s, the tissue was subjected to an ice bath for 30 min and centrifugation at 14200 g for 15 min. The supernatant was taken for testing.

The total protein concentration of each sample was determined with a BCA kit (PC0020-500, KeyGen Biotech Co., Ltd., Nanjing, China). The proteins were then separated by 12% sodium dodecyl sulfate-polyacrylamide gel electrophoresis (SDS-PAGE) and transferred to a polyvinylidene fluoride (PVDF) membrane (MilliporeSigma, St. Louis, MO, USA). After blocking with 5% nonfat milk powder (lp0031, OXOID, Hampshire, UK) diluted in Tris-buffered saline for 2 h, the membranes were incubated overnight at 4°C with primary antibodies against *β*-actin (66009-1-lg, Proteintech, Chicago, IL, USA), KGF (orb10652, Biorbyt, Cambridge, UK), and KGFR (ab10648, Abcam, Cambridge, MA, USA). The membranes were washed three times with 1 × TBST buffer and incubated with 1 : 1000 diluted HRP-labeled sheep anti-rabbit secondary antibody (zb2305, Zhongshan Golden Bridge Biotechnology Co., Ltd., Beijing, China) at room temperature for 1 h, and ECL (Cat# CW0049, CWbio Co., Ltd., Beijing, China) chemical staining was performed. Immunoreactive bands were detected using ImageQuant LAS 4000 mini (GE, Boston, MA, USA). The targeted protein was analysed using the ImageQuant TL software.

The concentration of KGF in the supernatant of lung homogenate and the levels of IL-6 and IL-13 in serum, BALF, and intestinal mucus were determined according to the manufacturer's instruction of the ELISA kit (KGF:ELM-FGF7-1, RayBio, Norcross, GA, USA; IL-6: ab222503, Abcam; IL-13: ab219634, Abcam). The content was calculated according to the standard curve and OD value. The average value of the test results was taken for data statistics.

### 2.8. Real-Time Quantitative PCR (qPCR)

Total RNA in lung samples was extracted with the ultrapure RNA extraction kit (Cat# CW0581, CWbio Co., Ltd.) and was reverse-transcribed into cDNA using a HiFi-MMLV cDNA kit (Cat#CW0744, CWbio Co., Ltd.). The RNA expression levels were detected by Ultra SYBR Mixture (with ROX) (Cat#CW0956, CWbio Co., Ltd.) according to the manufacturer's instruction. PCRs were performed in Line Gene type 9600 Plus Real-Time PCR system (Bioer Technology Co. Ltd., Hangzhou, China). The primer sequences were as follows: *β*-actin (FW 5′-GCCTTCCTTCTTGGGTAT-3′ and RV 5′-GGCATAGAGGTCTTTACGG-3′) and KGF (FW 5′-TGCTTCCACCTCGTCTGTC-3′ and RV 5′-TCCTTCCATGTAGTCATAACTTCTG-3′). The amplification program was 95°C for 10 min followed by 45 cycles of 95°C for 15 s and 60°C for 60 s. The quantity of specific mRNA was normalized to the expression level of internal control *β*-actin mRNA. Gene expression was calculated using the 2^ΔΔCt^ method.

### 2.9. Isolation of *γδ*T Lymphocytes and Flow Cytometer

With reference to the currently widely used and reliable method for separation of small intestinal intraepithelial lymphocytes, our research group established a centrifugation method of separation and purification of lymphocytes of lung tissue [14–22]. The whole lung tissues of mice were removed and cleaned with PBS (SH30256.01, Hyclone, South Logan, UT, USA) and then mechanically cut into tiny pieces by scissors. The pieces were flushed on a 70 *μ*m cell strainer (352350, Corning, NY, USA) and then collected into 50 ml tubes containing 10 ml of digestion solution which consisted of RPMI-1640 (22400-089, GIBCO, Grand Island, NY, USA) with 10% FBS (1600-044, GIBCO, Grand Island, NY, USA), 2 mg/ml Collagenase NB4 (17465.02, SERVA Electrophoresis, Heidelberg, Germany), and 40 *μ*g/ml DNase (DN25, Sigma-Aldrich, St. Louis, MO, USA). The digestion was at 37°C for 90 min, and the tubes were shaken once every 10 min. The solution was passed through a 70 *μ*m cell strainer and then centrifuged (Hitachi, Tokyo, Japan) at 374 g for 10 min to remove the supernatant. The cells were resuspended in Hanks' balanced salt solution (HBSS) (14175-095, GIBCO, Grand Island, NY, USA) containing 5% FBS and 30% percoll (17-0891-01, GE Healthcare, Uppsala, Sweden) and then centrifuged at 337 g for 18 min. The supernatant was discarded, and the cells were resuspended in the 1 ml left. The cell suspension was mixed in 10 ml of 44% isotonic percoll and underlaid by 2 ml of 70% isotonic percoll. After centrifugation at 337 g for 18 min, the viable cells were collected from the 44%/70% interface areas, some epithelial cells were at the top of the gradient, while the erythrocytes and debris were at the bottom. 1/3 of the top layer of the gradient and the cells coat at the interface were removed successively by vacuum aspiration. After washed by centrifugation in HBSS containing 5% FBS, the cell suspension in RPMI1640 containing 10% FBS was prepared to be measured. The number of cells was estimated by counting in a hemocytometer using trypan blue. Cells were stained with FITC anti-*γδ*TCR (ab118864, Abcam, Cambridge, MA, USA) and PE anti-*αβ*TCR (ab25649, Abcam, Cambridge, MA, USA) and then detected by FACS Calibur flow cytometer (FACS Calibur, BD, San Jose, CA, USA).

### 2.10. Statistical Analysis

The data were expressed as the mean ± standard error. SPSS 20.0 statistical software (IBM, Armonk, NY, USA) was used for statistical analysis. Normality test was first performed, and *t*-test or nonparametric Mann–Whitney *U* test was used to compare groups individually. The significant *p* value was calculated, and *p* < 0.05 was considered as significantly different.

## 3. Results

### 3.1. Life Quality and Body Weight

**Figure 1:**
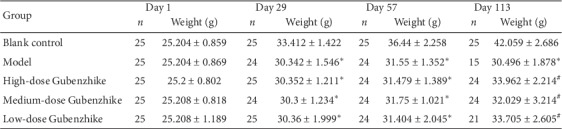
Gubenzhike recipe increased the survival rate and body weight of mice with COPD. Data are presented as mean ± SD. None of the blank control group died. They had smooth fur and gentle breath in moderate frequency and uniform rhythm. Mice of the blank control group behaved normally and gradually gained weight. 10 of the COPD model group died. Fur of mice in the model group was dry and dark, some of which lost. Mice moved and jumped restlessly when exposed to cigarette smoke. In the later stage, they often huddled and curled up, somewhile their whole body trembled. They breathed rapidly even with their mouths open. Their chest and abdomen undulated obviously, and they sometimes nodded irregularly. Body weight of mice with COPD was significantly lower than that of the blank control group (^*∗*^*P* < 0.001). High-dose and medium-dose Gubenzhike recipe groups had 1 death, respectively, while the low-dose group had 4. Compared with the model group, mice in three Gubenzhike recipe groups had softer and shinier fur, less agitation, slower respiratory rate, and significantly heavier weight (^#^*P* < 0.001).

After four months' experiment, mice in the blank control group did not die. They had burnished fur, normal breathing, and gradually increased weight. During 12 weeks of continuous exposure to cigarette smoke and LPS instillation in the respiratory tract, mice in the other four groups gradually lost the luster of their fur and had dyspnea, and their weight was significantly lower than the healthy ones. Gubenzhike recipe improved life quality of mice with COPD, made them have softer and shinier fur, less agitation, and slower respiratory rate, and obviously increased their weight (Figure 1).

### 3.2. Gubenzhike Recipe Improved the Pulmonary Histopathological Changes of COPD Model Mice

**Figure 2:**
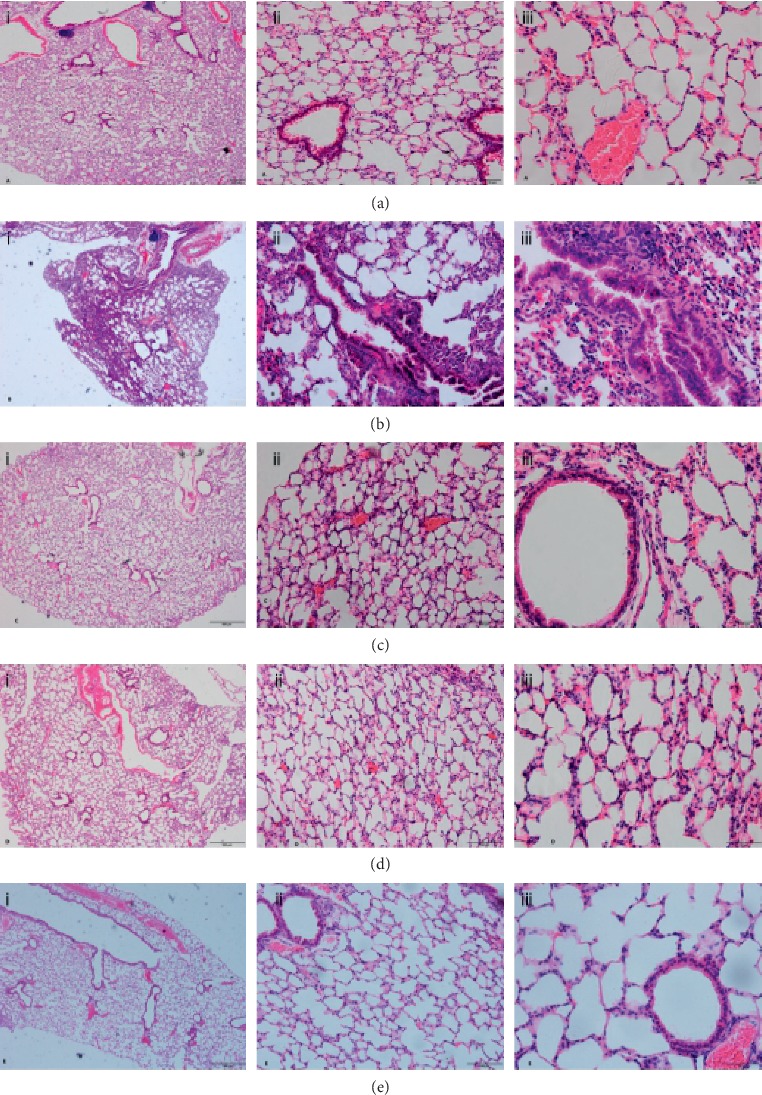
Three months after cigarette smoke and LPS challenge, lung pathology was determined by H&E staining (A: blank control group, B: model group, C: high-dose Gubenzhike recipe group, D: medium-dose Gubenzhike recipe group, and E: low-dose Gubenzhike recipe group). The marks i, ii, and iii stand for magnification of 40, 200, and 400, respectively, and their scale bars are equal to 200 *μ*m, 50 *μ*m, and 20 *μ*m. HE staining showed that the bronchial and alveolar structures of the blank control group were normal and clear, with orderly cilia, uniform alveolar size, and complete airway epithelium (Figure 2(a)). In the model group, the bronchial mucosal epithelium folds, exfoliated, and protruded into the lumen, which narrowed and occluded the bronchi. The alveolar wall collapsed, the alveolar cavity was irregularly enlarged, and part of the alveoli fused into lung bullae. The airway wall and pulmonary mesenchyme were infiltrated with chronic inflammatory cells (Figure 2(b)). These were consistent with the pathology of COPD. Gubenzhike recipe significantly increased the structural completion of the bronchi, alveoli, and airway mucosa epithelium. The arrangement of cilia was more regular and alveolar size was evener. Gubenzhike recipe also decreased the stenosis degree of airway, the number of lung bullae, and the number of inflammatory cell infiltrated around the airway and pulmonary mesenchyme (Figures 2(c)–2(e)).

The pathological manifestations of COPD include degeneration, necrosis, and detachment of bronchial mucosa epithelium, with the cilia being shorter and adhered. The trachea cavity becomes narrow because of hypertrophy of goblet cells and mucous cells, retention of abundant mucous, airway wall congestion, and edema. The alveoli atrophy and collapse, the alveolar septa is destroyed and ruptured, and adjacent alveoli fuse to form large alveoli. A large number of chronic inflammatory cells infiltrate around the trachea and alveoli. There are bronchi remodeling, increased collagen content, and scar formation (Figure 2(b)).

Gubenzhike recipe significantly improved the morphology situation of COPD mice lungs. It increased the structural integrity of the bronchi, alveoli, pulmonary mesenchyme, and the orderliness of cilia. Gubenzhike recipe also decreased the stenosis degree of airway, the number of lung bullae, and the number of inflammatory cells infiltrated around airway and pulmonary interstitium (Figures 2(c)–2(e)).

### 3.3. Gubenzhike Recipe Reduced the Degree of Airway Obstruction and Improved the Compliance of Lung Tissue

**Figure 3:**
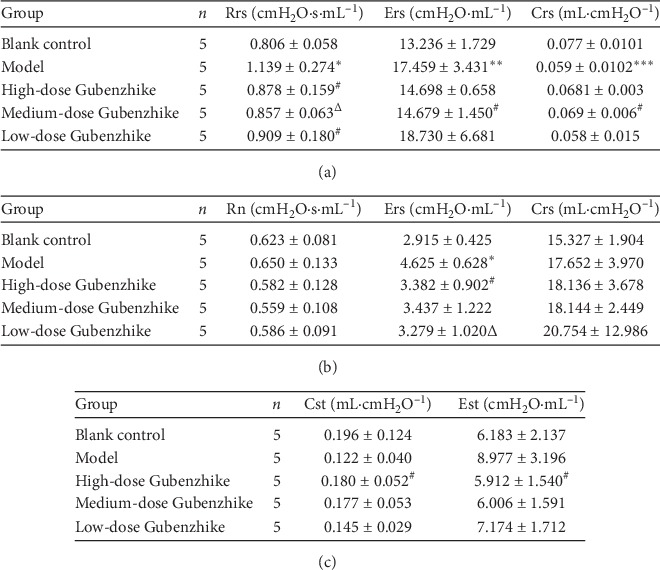
(a) Lung function parameters: the resistance of the respiratory system (Rrs), elastance of the respiratory system (Ers), and compliance of the respiratory system (Crs). Data are presented as mean ± SD. Compared with that of the blank control group, the Rrs and Ers of the model control group were significantly higher (^*∗*^*P*=0.009 < 0.05,^*∗∗*^*P*=0.039 < 0.05), and the Crs was significantly lower (^*∗∗∗*^*P*=0.024 < 0.05). Compared with that in the model control group, the Rrs of three Gubenzhike recipe groups was significantly lower (^#^*P*=0.047 < 0.05,^Δ^*P*=0.028 < 0.05), the Ers of the medium-dose Gubenzhike recipe group was significantly lower (^#^*P*=0.047 < 0.05), and the Crs was significantly higher (^#^*P*=0.047 < 0.05). (b) Lung function parameters: Newtonian resistance (RN), tissue damping (G), and tissue elastance (H). Data are presented as mean ± SD. Compared with G of the model group, that of the blank control group was significantly lower (^*∗*^*P*=0.009 < 0.05), and that of the high-dose and low-dose Gubenzhike recipe group was significantly lower (^#^*P*=0.009 < 0.05,^Δ^*P*=0.036 < 0.05). (c) Lung function parameters: quasistatic elastance (Est) and quasistatic compliance (Cst). Data are presented as mean ± SD. Compared with that of the model group, the Est was significantly higher and the Cst was significantly lower in the high-dose Gubenzhike recipe group (^#^*P*=0.047 < 0.05).

Compared with the blank control group, the lung function of the model group decreased, according to the increased obstruction and decreased dynamic compliance, which was consistent with the pathological changes of COPD (Figure 3). This suggests that cigarette smoke exposure can increase the degree of airway obstruction and decrease tissue compliance in mice. Gubenzhike recipe decreased tissue resistance and degree of airway obstruction and improved tissue compliance and lung function.

### 3.4. Gubenzhike Recipe Reduced the Ratio of *αβ*T Cell/*γδ*T Cell in Lungs of Mice with COPD Detected by Immunohistochemistry and Flow Cytometry

In normal mouse lungs, most *αβ*T cells are located in the parenchyma/alveoli and most *γδ*T cells are located in nonalveolar areas except the mucosa. The relative density of *γδ*T cells was the highest near the airways, blood vessels, and visceral pleura. Although their number is much smaller, the relative density of *γδ*T cells matches or nearly matches that of *αβ*T cells in nonalveolar areas but is much lower in the parenchyma area of which tissue surface is the largest. The difference in the distribution of these two T cells may be related to different functional roles. The narrow regional distribution of *αβ*T cells in the normal lung may reflect some degree of functional homogeneity, while the wide distribution of *γδ*T cells may reflect functional heterogeneity [23].

**Figure 4:**
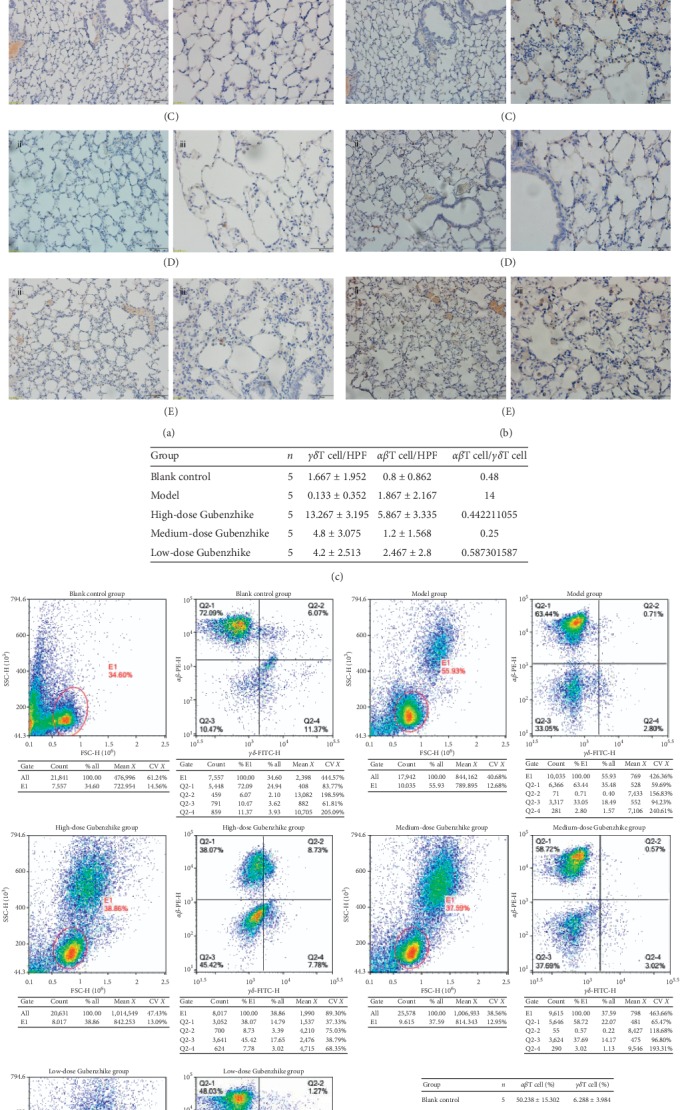
Immunohistochemical staining of *αβ*TCR (a) and *γδ*TCR (b) was made, respectively, on the paraffin lung sections (A: blank control group, B: model group, C: high-dose Gubenzhike recipe group, D: medium-dose Gubenzhike recipe group, and E: low-dose Gubenzhike recipe group). The marks ii and iii stand for magnification of 200 and 400, respectively, and their scale bars are equal to 50 *μ*m and 20 *μ*m. In the blank control group, there were a few *αβ*T cells and *γδ*T cells in the lung tissue. As for the model group, scattering distribution of *αβ*T cells in the alveolar tissue and hardly any *γδ*T cells were observed. There were significantly more *γδ*T cells around bronchi and rarefied *αβ*T cells in the three Gubenzhike recipe groups. (c) Positive brown cells were counted in three random fields at 400x magnification in each section with immunohistochemical staining of *αβ*TCR and *γδ*TCR. Data are presented as mean ± SD. The ratio of *αβ*T cell/*γδ*T cell reduced in lung tissue of COPD model mice and increased by Gubenzhike recipe. (d) The lymphocytes in lung tissues were isolated through density gradient centrifugation method and then analysed by flow cytometry. Gate E1 identified lymphocytes among total. Percentage of *αβ*T and *γδ*T lymphocytes was recorded, and data are presented as mean ± SD. The percentage of *γδ*T lymphocytes in the low-dose Gubenzhike recipe group was significantly higher than that in the model group (^&^*P*=0.012 < 0.05). Compared with the blank control group, the ratio of *αβ*T/*γδ*T lymphocytes in all other groups increased. That ratio decreased in high-dose and low-dose Gubenzhike recipe groups in contrast to that of the model group.

*γδ*T cell is a key effector cell of the respiratory mucosal immune system and participates in the process of inflammation and injury repair in chronic inflammatory diseases. We explored the method of separation of lymphocyte in lung tissue through density gradient centrifugation based on the separation method of lymphocyte between intestinal epithelial cells, and the *γδ*T cells in mice lungs were isolated for the first time. In this study, we found that the ratio of *αβ*T cell/*γδ*T cell in the model group was higher than that in the blank control group and lower than that in the Gubenzhike recipe group (Figure 4).

### 3.5. Gubenzhike Recipe Increased the Content of KGF and KGFR in Lung Tissues of Mice with COPD

The keratinocyte growth factor (KGF) is closely related to epithelial wound healing. Combined with keratinocyte growth factor receptor (KGFR) on epithelial cells, KGF can repair damage and maintain the integrity of epithelium, restrict pulmonary epithelial permeability and airway inflammation to prevent pulmonary edema, and inhibit the progression of pulmonary fibrosis.

**Figure 5:**
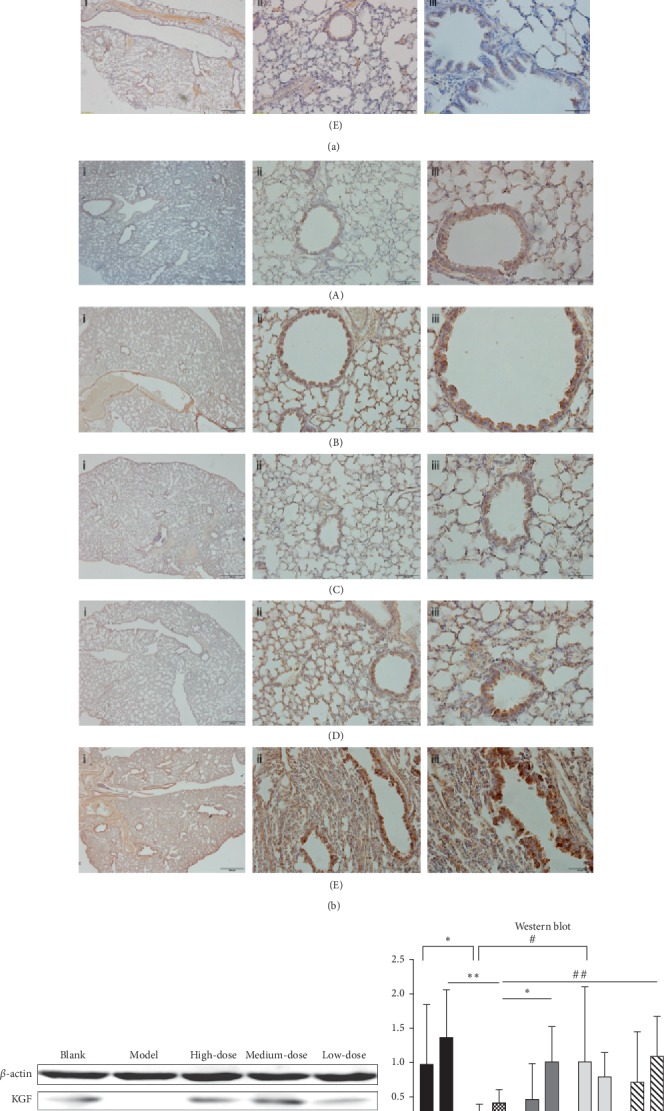
(a) Gubenzhike recipe increased KGF content in lung tissues of mice with COPD. Immunohistochemical staining of KGF and KGFR was made, respectively, on the paraffin lung sections (A: blank control group, B: model group, C: high-dose Gubenzhike recipe group, D: medium-dose Gubenzhike recipe group, and E: low-dose Gubenzhike recipe group). The marks i, ii, and iii stand for magnification of 40, 200, and 400, respectively, and their scale bars are equal to 200 *μ*m, 50 *μ*m, and 20 *μ*m. In the blank control group and model group, there were a few brown particles representing little expression of KGF. Large area of brown particles was visible in three Gubenzhike recipe groups, in which KGF expression was significantly higher. (b) Gubenzhike recipe increased KGFR content in lung tissues of mice with COPD (A: blank control group, B: model group, C: high-dose Gubenzhike recipe group, D: medium-dose Gubenzhike recipe group, and E: low-dose Gubenzhike recipe group). The marks i, ii, and iii stand for magnification of 40, 200, and 400, respectively, and their scale bars are equal to 200 *μ*m, 50 *μ*m, and 20 *μ*m. The amount of brown particles was more in the model group and three Gubenzhike recipe groups than the blank control group. (c) Three months after cigarette smoke and LPS challenge, lungs were collected. The protein levels of KGF and KGFR were analysed by western blot and ELISA according to the manufacturer's instructions. The mRNA level of KGF in lung tissues was examined by real-time PCR. Data are presented as mean ± SD, *n* = 5 in each group. Compared to that of the model group, KGF was significantly higher in the blank control group (^*∗*^*P*=0.047 < 0.05) and medium-dose Gubenzhike recipe group (^#^*P*=0.028 < 0.05), and KGFR was significantly higher in the blank control group (^*∗∗*^*P*=0.02 < 0.05), high-dose Gubenzhike recipe group (^*∗*^*P*=0.047 < 0.05), and low-dose Gubenzhike recipe group (^##^*P*=0.041 < 0.05) in the western blot detection. ELISA results showed that KGF of the high-dose Gubenzhike recipe group was significantly higher than that of the model group (^*∗*^*P*=0.009 < 0.05). As for the RT-PCR assays, mRNA level of KGF was significantly higher in the blank control group (^*∗∗*^*P*=0.013 < 0.05), medium-dose Gubenzhike recipe group (^###^*P*=0.018 < 0.05), and low-dose Gubenzhike recipe group (^*∗∗∗*^*P*=0.04 < 0.05) than in the model group.

In our study, we found KGF expression reduced in the lungs of mice with COPD than normal lungs. Gubenzhike recipe promoted the secretion of KGF which was abundant around the epithelium to repair lung tissue injury (Figures 5(a) and 5(b)). The results of western blot, ELISA, and RT-PCR analysis showed that KGF and KGFR protein and KGF mRNA in the model group were significantly lower than those in the blank control group and were increased by Gubenzhike recipe (Figure 5(c)).

### 3.6. Gubenzhike Recipe Increased IL-6 in Serum and BALF and IL-13 in BALF and Intestinal Mucus of Mice with COPD

**Figure 6:**
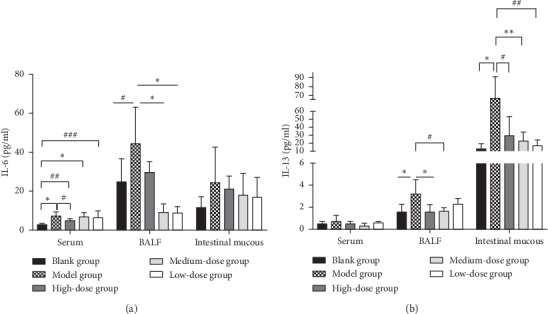
The secretion of inflammatory factors in mice with COPD was significantly increased and was reduced by Gubenzhike recipe. The levels of IL-6 and IL-13 in serum, BALF, and intestinal mucus of the mice were determined by ELISA according to the manufacturer's instructions. The IL-6 level in serum of the blank control group was significantly lower than that of the other four groups (^*∗*^*P*=0.002 < 0.05,^##^*P*=0.009 < 0.05,^###^*P*=0.016 < 0.05), while the content of IL-6 in the high-dose Gubenzhike recipe group was significantly lower than that in the model group (^#^*P*=0.028 < 0.05). The IL-6 level in BALF of the model control group was significantly higher than that of the blank control group (^#^*P*=0.049 < 0.05) and the medium-dose and low-dose Gubenzhike recipe groups (^*∗*^*P*=0.003 < 0.05). There was no significant statistical difference in IL-6 level of intestinal mucus and IL-13 level of serum between the groups. The IL-13 level in BALF of the model group was significantly higher than that of the blank control group (^*∗*^*P*=0.033 < 0.05) and the high-dose and medium-dose Gubenzhike recipe groups (^*∗*^*P*=0.033 < 0.05;^#^*P*=0.047 < 0.05). The IL-13 level in intestinal mucus of the model group was significantly higher than that of the blank control group (^*∗*^*P*=0.007 < 0.05) and the high-dose, medium-dose, and the low-dose Gubenzhike recipe groups (^#^*P*=0.041 < 0.05,^*∗*^*P*=0.006 < 0.05,^##^*P*=0.008 < 0.05).

IL-6 levels increased in serum and BALF while IL-13 levels increased in BALF and intestinal mucus of mice with COPD, and all of these were significantly reduced by Gubenzhike recipe, suggesting that systemic and local airway inflammatory response existed and was reduced by Gubenzhike recipe (Figure 6).

## 4. Discussion

### 4.1. Gubenzhike Recipe Can Enhance the Defensive Function of Vital Qi of Patients with Stable COPD and Improve Their Immunity

COPD belongs to the category of lung distension and syndrome characterized by dyspnea in traditional Chinese medicine (TCM) because the typical clinical symptoms of it are dyspnea, cough, and sputum production. Patients mainly have lung disease in TCM at the early stage of COPD. As qi and Yin were consumed in the long course of the disease, deficiency of spleen and kidney and other organs arose. It was difficult for body to remove the pathogens and recover vital energy, leading to a vicious circle and lung distension characterized by panting breath, large amount of phlegm, palpitation, and edema. TCM pathogenesis of stable COPD is characterized by deficiency of lung, spleen, and kidney, phlegm-turbid retention, and deficiency complicated with excessiveness but mainly deficiency. Lung in TCM is in charge of the vital qi and respiratory function. Invasion of exogenous pathogenic factors would lead to the failure of the lung to disperse and descend qi. Rebellion of lung qi would cause cough and qi deficiency would cause short breath. Kidney in TCM governs reception of qi, so breathlessness will appear if kidney fails to receive qi. In TCM theory, lung regulates water while spleen transports and transforms liquid. Body fluid would assemble and turn into stasis and phlegm because of the dysfunction of lung and spleen [24].

The treatment principle of COPD acute stage is to dispel pathogenic factors, while stable stage is primarily to reinforce deficiency according to the main disease location. On the basis of traditional Chinese medicine theory and years of clinical experience, Professor Chao Enxiang summed up the method of regulating lung and kidney, invigorating qi and strengthening spleen, as well as clearing heat and eliminating phlegm, activating blood circulation, and removing blood stasis, so as to restore the physiological function of “wei qi.” After clinical treatment for patients with stable COPD, the symptoms of cough, phlegm, asthma, and airway hyperresponsiveness were significantly improved, the number of acute exacerbation was reduced, and the immune capacity and the quality of life were enhanced [24, 25].

The prescription of Gubenzhike recipe is created based on Professor Chao Enxiang's experience of clinical work which is more than 60 years. Astragalus is sweet and gentle in the herb property of TCM, and it can help to invigorate qi and strengthen the resistance of body and tonify lung and spleen. Epimedium is also sweet and gentle in the herb property of TCM, and it can help to warm Yang and tonify kidney. Two herbs above are the monarch drug of the prescription. Rhizoma Atractylodes is bitter and sweet in the herb property of TCM, and it can help to invigorate spleen to supplement qi and transmit dampness. Honey-fried Radix Stemonae is sweet and bitter in the herb property of TCM, and it can help to warm and humidify qi of lung and descend the adverse flow of qi to relieve cough. These two herbs are the minister drug of the prescription. Radix Paeoniae Rubra is bitter and slightly cold in the herb property of TCM, and it can help to clear heat to cool blood and activate blood to remove stasis. Radix Scutellariae is bitter and cold in the herb property of TCM, and it can help to clear heat and dry dampness. Radix Saposhnikoviae is acrid and sweet and slightly warm in the herb property of TCM, and it can help to dispel the wind. Three herbs above are the guide drug of the prescription. All the herbs together can strengthen the patient's vital qi and dispel the invading pathogenic factors, supplement qi and warm yang, invigorate spleen and kidney, relive cough and reduce phlegm, activate blood, and remove stasis and thus enrich the vital energy and prevent aggravation [24].

The Gubenzhike capsule made from this prescription was used to treat the patients of chronic bronchitis at protraction stage with the TCM syndrome of deficiency in both lung and kidney and obstruction in the lung by phlegm. Clinical observation showed that Gubenzhike capsule had good efficacy in improving cough, expectoration, and wheeze, and the overall effective rate was 96.67%. In addition, it improved lung function and regulated immune function [8].

### 4.2. The Barrier Defense Effect of “Fei (Lung) Wei (Defense)” in TCM Theory Is Correlated with Mucosal Immune Function

#### 4.2.1. The Immune Function of Modern Medicine Is the Same as the “Vital Qi” Function of TCM Theory

Immunity in modern medicine refers to the body's own defense mechanism, which includes the recognition and clearance of foreign invasion substances, the dealing with aged, injured, died, and degenerated cells, and the identification and treatment of mutated and infected cells in the body. This immune function can be regarded as a concrete embodiment of the function of qi in TCM theory. The theory of Plain Questions (SuWen) says that “if vital qi is kept inside, the pathogens would not disturb.” TCM doctors believe that the low immune function is related to the lack of vital qi and its defensive function. In terms of concept and function, there are many similarities between the theory of vital qi in TCM and immunity. They are both normal physiological functions of the body, which jointly play the roles of defense, self-stabilization, and surveillance. “Vital qi dispels evil” means to drive away pathogens, which is similar to the defense function of the immune system to remove pathogenic antigens. The role of vital qi in regulating the balance of Yin and Yang is similar to self-stabilizing function of the immune system. Vital qi coordinates the organs, regulates qi and blood, and harmonizes Yin and Yang without phlegm and stagnation of blood stasis, which is similar to the surveillance function of the immune system [26, 27].

#### 4.2.2. There Are Similarities between Wei Qi and Mucosal Immunity in Location, Nature, and Mechanism of Action

Wei qi belongs to the vital qi. It has functions of warming the muscles, moistening the skin, and regulating striae. It spreads all over the body and supports the defense network, depending on the dispersing of lung qi. If wei qi is strong, the body's defense function can play a role in resisting the invasion of external pathogens. If qi is deficient in the lung, then body barrier will lose its firmness. Therefore, lung and qi are so closely related to each other as to be called “fei (lung) wei (defense),” which can protect the body and is similar to the mucosal immune function [28].

Mucosal immune system (MIS) consists of local mucosa-associated lymphoid tissue and diffuse lymphoid tissue. Human mucosal immune cells account for 80% of all immune cells, so mucosal immunity plays a very important role in the immune system. The first similarity between wei qi and mucosal immunity is position and structure. The surface area of human mucosa is about 400 m^2^, covering the gastrointestinal tract, respiratory tract, urogenital tract, and some exocrine glands. It is the main portal for pathogenic microorganisms and other harmful foreign matters to invade the body and plays an important role in defense and resistance. Wei qi is the outermost defensive qi of the human body in the theory of TCM. It moves around the skin to protect the body against the invasion of external pathogens and thus can be regarded as the first barrier of human body. This provides a basis for the correlation between mucosal immunity and “*fei (lung) wei (defense)*.” From the perspective of composition, the immune system includes humoral immunity and cellular immunity in addition to mucosal immunity and forms a complex immune network by communicating and cross-linking between various lymphocytes and cytokines. Mucosal immunity is also a network structure and composed of lymphoid tissue, immunoglobulin, and lysozyme. According to the theory of TCM, vital qi includes a variety of “qi” in addition to wei qi, whose distribution and circulation in the human body are also network like. The connection between the local immune network and the whole immune network depends on the presentation, which coincides with the relationship of wei qi and vital qi [29–31].

Wei qi and mucosal immunity also have similarities in nature. First of all, both of them have “quick response.” When the harmful substances from the outside invade the human body, they first pass through the skin and mucosa. Therefore, mucosal immunity responds early and fast which has something in common with the characteristic of rapid and fluent movement of wei qi. Secondly, both of them are “swimming.” The mucous blanket formed by the cilia and secretions on the mucosa and the immune cells under the mucosa are constantly swimming, which makes the local immune function systematic at the same time. Wei qi moves in Yang in the daytime and in Yin at the night time, round and round and back and forth in the body. Finally, both of them have “tropism.” When allergens invade the mucosa, lymphocyte, cytokines, and complement of submucosal lymphoid tissue will accumulate in the invaded site to participate in the immune response. When the body is affected by foreign factors, wei qi in the circulation will also move to the invaded site to prevent from further invading [26, 30].

Wei qi and mucosal immunity are related in the mechanism of action. The uptake and transport of antigen by M cells in mucosal immune response are similar to the control of matter moving in and out through the skin of wei qi. Antigen-presenting cells (APCs) and dendritic cells (DC) can initiate immune cascade reaction; however, when wei qi is not enough to resist, it will arouse a variety of “qi” in the body, such as ying qi, pi (spleen) qi, and fei (lung) qi, to fight against external pathogens. These two reactions are similar. *γδ*T cells in mucous membrane can directly kill infected cells, remove dead cells and foreign bodies, and resist tumors. The nonspecific natural immune function is similar to the function of dispelling external pathogens of wei qi. The important physiological functions of mucosal epithelial cells, such as nutrient absorption, gas exchange, secretion, and transport, are similar to the functions of wei qi of warming and nourishing the viscera and muscles, regulating sweat excretion, and controlling body barrier to open or close. Wu suggested there were close relations between generation of wei qi and “marrow” on the basis of “wei qi is rooted in the lower energizer,” “kidney is in the lower energizer,”nand “kidney controlling bones and production of bone marrow” in The Yellow Emperor's Canon of Internal Medicine. Leukocyte and mononuclear macrophages are derived from bone marrow pluripotent stem cells, and these nonspecific immune cells may be the material basis of the wei qi. The number of Th cells and sIgA level decreased in patients with fei (lung) qi deficiency and pi (spleen) qi deficiency and increased in patients after taking qi tonic drugs, indicating that the cells and immune molecules involved in mucosal immunity are the material basis of wei qi. The above demonstrates the relationship between wei qi and mucosal immunity from multiple perspectives [30, 32].

### 4.3. The Mechanism of TCM's Strengthening of Wei Qi Is to Enhance the Mucosal Immune Barrier and Regulate Mucosal Immune Cells and Factors

Traditional Chinese medicine can enhance the stability of the mucous membrane, maintain the integrity of intestinal mucosa tissue, reduce excessive intestinal mucosa permeability, promote intestinal peristalsis, and increase intestinal mucosal blood flow. It can also reduce the apoptosis of mucosal cells, promote the apoptosis of lymphocytes, downregulate the immune response, and protect the colon mucosa [32].

SIgA is a type of IgA which mainly exists in the mucosal tissue. It can prevent bacteria and viruses from adsorption to epithelial cells, eliminate bacteria, neutralize virus, inhibit virus replication, and reduce tissue damage caused by inflammation, thus protecting local mucosa. Traditional Chinese medicine can increase the secretion of sIgA, thus enhancing the immune function of mice's airway and intestinal mucosa. Liu et al. found that Shenlingbaizhu granules (a decoction for invigorating qi) could significantly increase sIgA level in saliva in children with repeated respiratory tract infection. It was inferred that sIgA was the material basis of the function of “wei qi” [26, 32].

The content of TNF-*α* and IFN-*γ* in the supernatant of PPL (lymphocytes of Peyer's patch) culture could be increased by both ginseng polysaccharide and polyporus polysaccharide. The latter also increased TNF-*α* and IFN-*γ* levels in the supernatant of IEL (intraepithelial lymphocyte) culture. Sijunzi decoction can increase the expression of TGF-*β* in the jejunum, reduce the expression of TNF-*α*, balance the TGF-*β*/TNF-*α*, and improve the Th1/Th2 cytokines, thereby regulating intestinal immunity, reducing local inflammatory response, and affecting the overall immune function of the body. Cheng found that after taking Sijunzi decoction, the basal anal temperature, serum d-xylitol content, intestinal SDH activity, and levels of IL-2 and IL-4 in intestinal mucosa of rats with spleen deficiency were significantly increased, while LDH activity and levels of IL-1*β* and IL-6 in intestinal mucosa were significantly decreased, which were dose-dependent. The results showed that traditional Chinese medicine could affect the activity of mucosal immunity through regulation of enzymatic activity and cytokines [26, 32].

Previous experiments of our research group showed that Gubenzhike recipe could reduce the secretion of IL-17 and the airway injury-repair process by adjusting the biological activity of *γδ*T cells. Increased secretion of KGF content of lungs was observed in immunochemical staining and ELISA. We suggested Gubenzhike recipe could promote the repair of lung tissue damage in mice with COPD, protect the integrity of respiratory mucosa, and enhance the mucosal immune function [33–37].

In conclusion, traditional Chinese medicine can enhance the mucosal immune function by protecting the mechanical mucosal barrier, regulating the number of cells in the mucosal immune-inducing and immune-effecting sites and their ability to secret cytokines, also balancing cytokines, and promoting the secretion of sIgA.

### 4.4. Mucosal Immunity Is the First Defense of the Respiratory System and *γδ*T Cell Is Involved in COPD

The mucosal tissue is in close contact with the external environment, and it is the earliest and biggest part of the body to be threatened by harmful substances. The defense system formed by the mucosal tissue and its related immune system can effectively prevent the occurrence of mucosal diseases. Although mucociliary clearing by cilia and mucus can prevent large volume material from entering alveoli, the respiratory mucosal immune system is required for clearance of small volume substance containing a large number of pathogens. According to the response process, the mucosal immune system can be divided into induction site and effective site: the induction site includes Peyer's patches, bronchus-associated lymphoid tissue (BALT), nose-associated lymphoid tissue (NALT), and other mucosa-related lymphoid tissues. The effective sites are mainly lymphocytes in lamina propria and intraepithelial lymphocytes. After activated at the induced site, T cells and B cells migrate to the effector site through the lymphocyte homing mechanism to perform their functions [38].

In addition to the underlying mesenchyme, pulmonary epithelium is considered to be the largest mucosal barrier to the environment. Hogg called the subepithelial lymphoid aggregates abundant in T and B cells “bronchus-associated lymphoid tissue,” amount of which was proportional to the severity of airflow limitations. *γδ*T cells, as the major components of this irregular lymphocytes pool, are common in epithelial cells. They connect innate and adaptive immunity and play a protective role in immune surveillance. T cells are major lymphocytes and play a critical role in cell-mediated immune response with T-cell receptor (TCR) expressed on the surface. *αβ*T cells of which TCR composed of alpha and beta chains make up the majority of T cells, while *γδ*T cells make up only a small portion. [6]

*γδ*TCR recognizes nonpeptide antigens such as glycerides and other small, soluble, or membrane-anchored peptides that are cross-linked to major histocompatibility complex molecules (MHC) or MHC-like molecules in an antigen-independent manner. *γδ*T cells monitor epithelial cells and form the first defense against infection and are also involved in tissue repair and regulation of epithelial homeostasis, consequently having a unique protective effect on the lung [11, 39, 40]. *γδ*T cells also play a role in inflammation, epithelial growth and repair, and prevention of epithelial malignancies. It has been shown that *γδ*T cells altered the development and function of myeloid cells, including dendritic cells (DCs) and macrophages in vitro [23]. In addition, they secrete cytokines and chemokines that recruit inflammatory neutrophils to speed up clearance of pathogens and repair damaged tissue [6, 41].

Being lack of *γδ*T cells in the skin can significantly delay the proliferation of damaged epidermal cells and wound healing [39]. Activated *γδ*T cells but not *αβ*T cells in the intestinal epithelium produced keratinocyte growth factor (KGF), so as to induce the proliferation and repair of epithelial cells and maintain the intestinal barrier. Nielsen found that mice lacking *γδ*T cells were more susceptible to DSS- (dextran sodium sulfate-) induced colitis, their tissue damage was much more serious, and their ability to repair damaged epithelial cells was worse [7, 40]. *γδ*T cells in the lungs, although small in number, can protect lung function and play an important role in the ability to recover from environmental stimuli. Most resident *γδ*T cells in the lung exist in nonalveolar areas except the mucosa. They seem to be extremely rare, but can monitor large numbers of pulmonary macrophages and DCs, which can help the host to defend against and improve pulmonary inflammation. *γδ*T cell population increased during pulmonary infection. They also regulate noninfectious pulmonary inflammation associated with injury or anaphylaxis and have been found to be associated with COPD [23]. Mroz published that the relative and absolute number of *γδ*T cells in induced sputum and BALF decreased in COPD patients compared to asthma patients and healthy individuals. The passivated response of *γδ*T cells in bronchoalveolar lavage fluid in patients with COPD has been described. Fewer *γδ*T cells were observed in COPD patients with lower FEV1, suggesting that *γδ*T cell is closely related to decreased lung function. The number of *γδ*T cells is inversely related to the number and time of smoking, supporting that *γδ*T cells are associated with protecting or repairing pulmonary damage caused by smoking [42–47].

In Urboniene's study, the number of *γδ*T cells had no significant difference in peripheral blood while decreased in induced sputum and BALF in COPD patients compared with asthma patients and healthy individuals. The *γδ*T cell number in lungs was not correlated with that in circulation. This indicated that the inflammatory response of *γδ*T cells occurred locally in the airway, not in the circulation. Previous studies have found that the primary subgroup of *γδ*T cells in the lung is V*δ*1 phenotype, and the primary subgroup of circulating *γδ*T cells is V*δ*2 phenotype, depending on their V gene. Differences in function and phenotype between pulmonary and circulating *γδ*T cells may explain why there is no correlation between the number of *γδ*T cells in peripheral blood and in lungs [42].

### 4.5. Gubenzhike Recipe Can Reduce the Inflammatory Response and Promote Tissue Repair of COPD by Upregulating the Proportion of *γδ*T Cell

According to the above research results, we decided to analyse *γδ*T cells in lung tissue. Intestinal intraepithelial lymphocytes (IELs) that mainly consist of T cells are resident in the basolateral side of intestinal epithelial cells (IECs). Different from that, pulmonary T lymphocytes are scattered in lung tissue, making it more difficult to isolate them. We have proved that density gradient centrifugation is a feasible method. It has been demonstrated that *γδ*T cell could help maintain the integrity of epithelial barrier and mucosal immunity in the lungs. *γδ*T cells are preferentially implanted in lung epithelial tissues to promote epithelial growth, regulate airway hyperresponsiveness through Th2 cells, and regulate inflammatory response during infection. Induction of traumatic pulmonary hemorrhage in mice resulted in an increase in the number of pulmonary *γδ*T cells which regulated *αβ*T cells and marrow-derived inhibitory cells into the pulmonary epithelium. Experimental studies have shown the protective effect of *γδ*T cells in tissue repair and respiratory infections or other agents of damage. Absence of *γδ*T cells leads to faster appearance of lung damage, more severe epithelial damage, and defect in removing damaged epithelial cells. Murdoch with colleagues reported that mice lacking *γδ*T cells showed delayed intraepithelial regeneration after allergen stimulation [11, 39, 40, 42, 48, 49].

We found there was a tendency that *γδ*T cell percentage decreased in the model group and increased in three Gubenzhike recipe groups. A tentative inference on this result is that Gubenzhike recipe can increase the ratio of *γδ*T cells in the lung tissue of mice with COPD to reduce inflammation, repair lung tissue damage, thus improve the pathological changes, maintain epithelial mucosa homeostasis, and enhance the mucosal immunity, so as to control the disease progression. A majority of patients with stable COPD have deficiency of lung and kidney and much likely other more organs due to long-term course and repeated acute exacerbations. Gubenzhike recipe can tonify lung and kidney, enhance wei qi, and restore its protective function, of which the mechanism we speculated was regulating the proportion of *γδ*T cells and enhancing mucosal immunity.

### 4.6. Gubenzhike Recipe Can Promote Tissue Repair in COPD by Promoting KGF Secretion

*γδ*T cells participate in the repair of intestinal, pulmonary, and corneal epithelial cells by producing KGF [6]. Keratinocyte growth factor (KGF), also known as the fibroblast growth factor 7 (FGF7), is a powerful factor that promotes epithelial cells mitosis and differentiation, almost entirely produced by cells of mesenchymal origin. By connecting to an alternative spliced tyrosine kinase receptor, FGF receptor 2 (FGFR2) type IIIb, KGF plays a major role in the epithelial cells. It has been reported that KGF can enhance the pulmonary innate immunity, inhibit the inflammatory response of epithelial cells, remove dead cells and bacteria in the alveoli, repair damaged epithelial cells, and help to improve emphysema.

Exogenous KGF has been published to protect the lungs of animals from various factors including hyperoxia, bleomycin, radiation exposure, LPS, bacteria, and mechanical ventilation damage. In various lung injury models in rodent, pretreatment of recombinant human KGF or pulmonary highly expressed KGF can reduce alveolar leukocyte infiltration, hemorrhage, pulmonary edema, permeability, hypoxia, epithelial injury, and fibrosis in lungs, improve tissue compliance, and increase the proliferation of type II alveolar/terminal bronchial epithelial cells [50]. KGF can stimulate the expression of surfactant proteins and phospholipids, maintain the expression of sodium ion channels after epithelial injury, and accordingly improve the barrier of alveolar type II epithelial cells (AECII). In addition, the protective effect of KGF is also related to the stimulation of ATII cell proliferation and differentiation, inhibition of apoptosis, reduction of DNA damage, and induction of reduced oxidative stress factors. The potential of KGF to protect epithelial and mucosal tissues was indicated by its action of reducing the severity of mucositis after chemoradiotherapy. Although the beneficial mechanism of KGF in mucositis is generally attributed to protecting the integrity of the epithelial barrier, Gardner and colleagues believed since infection was the central pathogenesis of the disease that KGF may have antibacterial effects [13, 51].

KGF may also enhance the innate immunity of the lungs. Viget reported that infusing KGF into the trachea of rats 48 hours before infection with *Pseudomonas aeruginosa* could improve the pulmonary barrier function, enhance bacterial clearance rate, and improve survival rate both in vivo and in vitro. Scientists have observed the reduction of bacterial translocation to the lung by KGF in *P. aeruginosa*-induced pneumonia and lung injury models and injured lung models by *E. coli* in vitro. Jason Gardner reported that recombinant human KGF (rhKGF) could induce alveolar epithelial cells to release granulocyte-macrophage colony-stimulating factor (GM-CSF), activate macrophages signal sensors and catalysts, and enhance the clearance of *E. coli*, *Pseudomonas aeruginosa*, and *Mycobacterium tuberculosis* in the lungs of mice. A study in vivo extended these findings to humans by demonstrating that BALF of subjects pretreated with recombinant human KGF enhanced the ability of alveolar macrophage (AM) to engulf bacteria through a GM-CSF-dependent mechanism. KGF in gingival fibroblasts was upregulated by LPS and inflammatory cytokines IL-1, IL-6, TNF, TGF, and platelet-derived growth factor BB. KGF stimulates the generation of antimicrobial peptides and increases the bactericidal activity of transplanted skin by 500 times, which all indicate that it can enhance the innate immune defense. In an artificial lung model with perfusion in vitro, KGF can increase alveolar fluid clearance and decrease the concentration of TNF-*α*, IL-1*β*, and IL-8 in BALF after LPS treatment. It has been shown that KGF enhanced the expression of pulmonary collectins surfactant protein SP-A and SP-D, which bind and aggregate bacteria, fungi, and viruses, directly activate macrophages, and enhance phagocytosis and intracellular killing of various pulmonary pathogens. SP-D is a marker of ATII cell proliferation. LPS stimulation and ARDS can cause the decrease of SP-D, while the low concentration of SP-D in alveolar edema fluid is associated with poor prognosis. Gardner et al. found that SP-A and SP-D directly inhibited the growth of Gram-negative bacteria through a membrane instability mechanism. Shyamsundar et al. first published a study in humans, which showed that intravenous therapy of palivmin increased SP-D in BALF, suggesting that it could reduce the damage of inhaled LPS to ATII type cells [50]. Studies on KGF knockout animals by Gardner et al. showed that insufficient KGF resulted in defective bacterial clearance and decreased phagocytosis of alveolar macrophages and antibacterial activity in BALF related to a decrease in levels of alveolar SP-A, SP-D, and lysozyme. It is speculated that KGF could support the innate immune defense of alveoli by maintaining the level of alveolar antibacterial protein and the function of alveolar macrophages, removing invading bacteria, reducing inflammatory factors, and enhancing lung barrier function [13, 50].

Our research showed that in contrast to that in the blank control group, expression of KGF and KGFR was significantly lower in the model group and increased to varying degrees in three Gubenzhike recipe groups. Therefore, we concluded that reduction of KGF and KGFR was one of the COPD pathogeneses. The delay of inflammation and harmful substances removal made injury difficult to heal and defensive ability reduce and then formed a vicious circle, which resulted in gradual aggravation. We theorized Gubenzhike recipe can promote the secretion of KGF and KGFR and removal of necrotic material and pathogens to inhibit epithelial inflammation, repair pulmonary injury, enhance pulmonary immunity, improve emphysema, and control disease progress, thus forming a protective effect on the tissue. These were assumed by us the experimental and theoretical basis for the function of Gubenzhike recipe to enhance vital qi.

### 4.7. Gubenzhike Recipe Can Reduce the Inflammatory Response in COPD by Downregulating the Chronic Inflammatory Cytokines

Progressive airflow limitation in COPD is caused by two major pathological processes: narrowing and remodeling of small airways and destruction of lung parenchyma with destruction of alveolar attachments in the airways due to secondary emphysema. The degree to which they affect the disease varies from person to person. Chronic inflammation around the lungs causes these pathological changes and accelerates the disease process. Small airway (diameter <2 mm) remodeling is characterized by an increase in airway wall tissue, including epithelium, lamina propria, smooth muscle, and the adventitia between the epithelial and muscular layers. These changes are related to the accelerated reduction of forced expiratory volume in the first second (FEV1). Matsuba and Thurlbeck initially assumed that airway remodeling leads to irreversible airflow limitations that prevent adequate dilation by bronchodilators and acetylcholine. In the course of COPD, airway remodeling is aggravated with increased mucus production and weakened mucociliary clearance, leading to the accumulation of mucus and inflammatory mediators in the lumen [12]. Obstruction of small airways and destruction of alveolar attachments result in airway closure, air trapping, and hyperinflation. Exercises will exacerbate these changes, leading to exertional dyspnea, the main symptom of COPD [2].

Inflammation in modern medicine refers to the process of tissue injury, congestion, swelling, exudation and degeneration, blood vessel destruction and necrosis or hyperplasia and embolism, local ischemia and hypoxia accompanied by metabolic function change, circulation disorder, blood flow variation, and so on. Inflammation can activate the body's immune system and is the body's automatic defense response. The essence of inflammation is a process of conflict between the injury caused by inflammatory factors and the body's anti-injury response, which has something in common with the TCM theory of struggling between vital qi and pathogenic factors. SIRS (systemic inflammatory response syndrome)/CARS (compensatory anti-inflammatory response syndrome) theory shows that SIRS is the protective response of body to external damage factors. SIRS and CARS constitute the two aspects of contradiction; they are a unity of opposites and change in reverse direction, which happens to coincide with the theory of vital qi and pathogenic factor TCM. After being invaded by pathogenic factors, the body produces inflammatory response (SIRS) and a series of anti-inflammatory responses (CARS). That is similar to “vital qi rising up to resist evil spirits” in TCM [52, 53].

From the perspective of modern medicine, COPD is not only a chronic inflammatory disease of the lungs, but also often accompanied by chronic diseases of multiple systems of the whole body. Some scholars believe that it may be a chronic systemic inflammatory syndrome. In recent years, some anti-inflammatory measures against SIRS, including hormones, monoclonal antibody of endotoxin and TNF-*α*, and other inflammatory mediators antagonists, have failed in clinical trials, and some of the tests even showed harmful effects, suggesting that we should not ignore the immunodeficiency status when CARS is dominant. To improve inflammation by regulating immunity, it is possible to balance injury and anti-injury on the whole, so as to promote vital qi and dispel pathogenic factors [53].

Cytokines are mediators of chronic inflammation. IL-6 is a multipotent cytokine that affects a variety of cell types and stimulates the expression of adhesion molecules and chemokines, thereby enhancing local inflammatory response by stimulating the recruitment of inflammatory cells [54]. IL-6 is consistently elevated in systemic circulation of patients with emphysema and COPD. Chiharu Tabata observed that IL-6 mRNA levels in the lung tissues of mice intervened by smoke extraction were significantly higher than those in the control group. Herfs M reported that anti-IL-6 therapy in vivo significantly reduced epithelial hyperplasia and squamous metaplasia which is associated with airway obstruction in patients with COPD and is a risk factor for tumor transformation and epithelial remodeling [55]. Elevated circulating IL-6 level is concerned with several complications, including ischemic heart disease, diabetes, and osteoporosis [2, 56].

It has been demonstrated that TH2 cytokine IL-13 participates in airway inflammation, promotes airway hyperresponsiveness, induces mucosal metaplasia and chemokine expression, and plays an important role in airway remodeling. In experiments and analysis of Janet S Lee's team, there was a positive correlation between IL-13 concentration in the cycle and the severity of airway obstruction and diffusion impairment, and a negative correlation between intracellular IL-13 and FEV1%. As shown in others' research, the proportion of IL-13 expressed by CD4+ and CD8+ T cells in BALF was significantly higher in the patients with COPD than smokers with normal lung function and characters who never smoke. It is highly likely that IL-13 is an important factor of COPD mechanism. Elias and colleagues published that overproduction of constitutive IL-13 in the lungs of adult mice induced pulmonary fibrosis, and overproduction of conditional lung-specific IL-13 leads to emphysema in IL-13 gene deficiency mice. IL-13 is also directly related to the airway mucus production by identifying signaling pathways activated in COPD involving IL-13R, calcium-activated chloride channel regulator1(CLCA1), and mitogen-activated protein kinase13 (MAPK13), which regulates the expression of MUC5AC gene in human airway epithelial cells [3, 57, 58].

We found that IL-6 in serum and BALF as well as IL-13 in BALF and intestinal mucus of mice with COPD decreased after Gubenzhike recipe intervention. Gubenzhike recipe reduced systemic inflammation involved IL-6 and airway inflammation in which both IL-6 and IL-13 participated. As a result of that, damage, obstruction, and mucus secretion of airway would decrease. We hypothesized Gubenzhike recipe can control the development of COPD and delay the formation of pulmonary fibrosis and emphysema through the mechanism mentioned above. Meiqin et al. showed that the addition of Peibenbufei powder on the basis of modern western medicine treatment could significantly improve the lung function and level of serum inflammatory factors, inhibit airway inflammatory response, and reduce inflammatory injury in patients with stable COPD. Gubenzhike recipe can enhance the vital qi to dispel pathogenic matters and strengthen the defensive function of wei qi which are similar to effects of reducing inflammatory cytokines and ameliorating inflammation. We considered those were experimental evidence for the mechanism of Gubenzhike recipe regulating immune system, treating COPD, regulating immunity, and playing a protective role in lungs of COPD.

## 5. Conclusion

Our research results suggest that Gubenzhike recipe reduced the content of IL-6 and IL-13, increased the proportion of *γδ*T cells in lung tissue, and promoted the secretion of KGF and KGFR and thus effectively controlled systematic and airway inflammation and improved the structure of airway and damage of lung tissue. The melioration of respiratory status and lung function of mice with COPD was notable. Function of Gubenzhike recipe to accelerate pulmonary tissue reparation, improve respiratory tract epithelial integrity, and enhance the respiratory mucosal immune function provided experimental evidence for its effects of reinforcing the defense, supervision, and self-stabilization function of “wei qi” by means of strengthening vital qi to eliminate pathogens, invigorating qi and Yang, tonifying spleen and kidney, relieving cough and reducing phlegm, activating blood, and removing stasis.

We would like to point out that mucosal immunity is a very complex system, and our study is only limited to *γδ*T cells in lung tissue and KGF they produce. However exploratory, this study may provide some insight into controlling the progression of COPD from the perspective of immunology and an experimental procedure for the isolation of *γδ*T lymphocytes from lung tissue.

## Figures and Tables

**Table 1:** Parameters of the lung function test.

Parameter	Description	Physiological relevance
Rrs	Resistance of the respiratory system	The degree of pulmonary obstruction
Ers	Elastance of the respiratory system	The elastic stiffness of the lungs
Crs	Compliance of the respiratory system	The ability to extend the lungs
Rn	Newtonian resistance	Central airway constriction
G	Tissue damping	Alveolar tissue constriction
H	Tissue elastance	Alveolar tissue stiffness
Cst	Quasistatic compliance	Ability to stretch
Est	Quasistatic elastance	The temporary blocking of airflow during the respiratory cycle (in inverse proportion to the elasticity of the lung tissue)

## Data Availability

All data generated or analysed in this study are included in this published article.

## References

[1] Vogelmeier C. F., Criner G. J., Martinez F. J. (2017). Global strategy for the diagnosis, management and prevention of chronic obstructive lung disease 2017 report. *Respirology*.

[2] Barnes P. J. (2014). Cellular and molecular mechanisms of chronic obstructive pulmonary disease. *Clinics in Chest Medicine*.

[3] Holtzman M. J., Byers D. E., Alexander-Brett J., Wang X. (2014). The role of airway epithelial cells and innate immune cells in chronic respiratory disease. *Nature Reviews Immunology*.

[4] Sato S., Kiyono H. (2012). The mucosal immune system of the respiratory tract. *Current Opinion in Virology*.

[5] Randall T. D. (2015). Structure, organization, and development of the mucosal immune system of the respiratory tract. *Mucosal Immunology*.

[6] Latha T. S., Reddy M. C., Durbaka P. V. R., Rachamallu A., Pallu R., Lomada D. (2014). Gammadelta T cell-mediated immune responses in disease and therapy. *Frontiers in Immunology*.

[7] Nielsen M. M., Witherden D. A., Havran W. L. (2017). *γδ* T cells in homeostasis and host defence of epithelial barrier tissues. *Nature Reviews Immunology*.

[8] Yin Y., Zhang H. C., Chao E. X. (2001). Clinical study on the treatment of chronic bronchitis with Gubenzhike capsule. *Journal of Beijing University of TCM*.

[9] Bove P. F., Dang H., Cheluvaraju C. (2014). Breaking the in vitro Alveolar type II cell proliferation barrier while retaining ion transport properties. *American Journal of Respiratory Cell and Molecular Biology*.

[10] Edelblum K. L., Singh G., Odenwald M. A. (2015). *γδ* intraepithelial lymphocyte migration limits transepithelial pathogen invasion and systemic disease in mice. *Gastroenterology*.

[11] McCarthy N. E., Eberl M. (2018). Human gammadelta T-cell control of mucosal immunity and inflammation. *Frontiers in Immunology*.

[12] Tam A., Sin D. D. (2012). Pathobiologic mechanisms of chronic obstructive pulmonary disease. *Medical Clinics of North America*.

[13] Gardner J. C., Wu H., Noel J. G. (2016). Keratinocyte growth factor supports pulmonary innate immune defense through maintenance of alveolar antimicrobial protein levels and macrophage function. *American Journal of Physiology-Lung Cellular and Molecular Physiology*.

[14] Bi J., Tong L., Zhu X. (2014). Keratinocyte growth factor-2 intratracheal instillation significantly attenuates ventilator-induced lung injury in rats. *Journal of Cellular and Molecular Medicine*.

[15] Anna C., Mañe J., Rebeca S. (2013). Comparison of lymphocyte isolation methods for endoscopic biopsy specimens from the colonic mucosa. *Journal of Immunological Methods*.

[16] Benno W., Tubbe I., Daniel S., Nicolaev A., Becker C., Neurath M. F. (2007). Isolation and subsequent analysis of murine lamina propria mononuclear cells from colonic tissue. *Nature Protocols*.

[17] Binda E., Erhart D., Schenk M., Zufferey C., Renzulli P., Mueller C. (2009). Quantitative isolation of mouse and human intestinal intraepithelial lymphocytes by elutriation centrifugation. *Journal of Immunological Methods*.

[18] Couter C. J., Surana N. K. (2016). Isolation and flow cytometric characterization of murine small intestinal lymphocytes. *Journal of Visualized Experiments*.

[19] Flano E., Jewell N. A., Durbin R. K., Durbin J. E. (2009). Methods used to study respiratory virus infection. *Current Protocols in Cell Biology*.

[20] Montufar-Solis D., Klein J. R. (2006). An improved method for isolating intraepithelial lymphocytes (IELs) from the murine small intestine with consistently high purity. *Journal of Immunological Methods*.

[21] Qiu Z., Sheridan B. S. (2018). Isolating lymphocytes from the mouse small intestinal immune system. *Journal of Visualized Experiments*.

[22] Ye Y., Yue M., Jin X., Chen S., Li Y. (2010). Isolation of murine small intestinal intraepithelial *γδ*T cells. *Immunological Investigations*.

[23] Wands J. M., Roark C. L., Aydintug M. K. (2005). Distribution and leukocyte contacts of *γδ* T cells in the lung. *Journal of Leukocyte Biology*.

[24] Chen Y. Y., Zhang H. C., Wu J. Q., Wang X. J. (2007). The train of thought and experience of Professor En-xiang Chao in the treatment of chronic obstructive pulmonary disease in stable period with the method of adjusting and supplementing lung and kidney. *Beijing Journal of TCM*.

[25] Guo Y., Wang X. Q. (2018). Professor En-xiang Chao's characteristics in diagnosis and treatment of chronic obstructive pulmonary disease. *Beijing Journal of Traditional Chinese Medicine*.

[26] Li J. Z., Tang X. L. Z., Fang C. T., Zhuang J. N., Chen T. (2019). Correlation of defensive Qi of traditional Chinese medicine with immune regulation and oncogenesis. *Journal of Oncology in Chinese Medicine*.

[27] Liu Z. D. (2013). Analysis of traditional Chinese medicine and immunology. *Chineese Archives of Traditonal Chinese Medicine*.

[28] Xu C. J., Xu X., He X. H. (2005). Identification of correlation between wei qi and mucosal immunity. *Chinese Archives of Traditonal Chinese Medicine*.

[29] Chen B. J., Yang M., Xu Y., Ming X., Xiong L. (2014). A brief discussion on the relationship between lung governing qi and mucosal immunity. *Journal of Nanjing University of TCM*.

[30] Chen Q., Zhang Q. X. (2014). Study on the correlation between wei qi and mucosal immune mechanism of allergic rhinitis. *China Journal of Traditonal Chinese Medicine and Pharmacy*.

[31] Panda S. K., Colonna M. (2019). Innate lymphoid cells in mucosal immunity. *Frontiers in Immunology*.

[32] Zhao H., Liu D. Y., Huang X. Y. (2009). Study on the regulation of mucosal immunity by traditional Chinese medicine. *Journal of Traditional Chinese Medicine*.

[33] Liu Y. J., Guo L., Shu J., Qin Y., Gu M. J. (2014). Effects of Gubenzhike Recipe on gamma delta-T cell and IL-17 in lung of COPD mice. *China Journal of Traditonal Chinese Medicine and Pharmacy*.

[34] Liu Y. J., Guo L. L., Shu J., Qin Y. Y., Gu M. J. (2015). Study of the effect of Gubenzhike recipe on the expression of keratinocyte growth factor in the lung of COPD mice. *World Journal of Integrated Traditional and Western Medicine*.

[35] Liu Y. J., Guo L. L., Shu J., Qin Y. Y., Gu M. J. (2015). Effects of Guben Zhike recipe on the spleen index, thymus index and Th1/Th2 unbalance in COPD mice. *China Journal of Traditonal Chinese Medicine and Pharmacy*.

[36] Lai X. C., Yu Q. S., Pan L., Zhao D., Zhang K. H. (2012). Effect of Gubenzhike recipe on pulmonary function and sIgA in respiratory tract of COPD mice. *China Journal of Traditonal Chinese Medicine and Pharmacy*.

[37] Lai X. C., Yu Q. S., Pan L., Zhao D., Zhang K. H. (2015). Effects of Gubenzhike recipe on expression of neutrophil elastase in the lung tissue of COPD mice. *China Journal of Traditonal Chinese Medicine and Pharmacy*.

[38] Wang J. T. (2015). Study progresses in respiratory mucosal immunity and related diseases. *China Journal of Immunology*.

[39] Murdoch J. R., Gregory L. G., Lloyd C. M. (2014). *γδ*T cells regulate chronic airway inflammation and development of airway remodelling. *Clinical & Experimental Allergy*.

[40] Fay N. S., Larson E. C., Jameson J. M. (2016). Chronic inflammation and gammadelta T cells. *Frontiers in Immunology*.

[41] Lawand M., Dechanet-Merville J., Dieu-Nosjean M. C. (2017). Key features of gamma-delta T-cell subsets in human diseases and their immunotherapeutic implications. *Frontiers in Immunology*.

[42] Urboniene D., Babusyte A., Lötvall J., Sakalauskas R., Sitkauskiene B. (2013). Distribution of *γδ* and other T-lymphocyte subsets in patients with chronic obstructive pulmonary disease and asthma. *Respiratory Medicine*.

[43] Vij N., Chandramani-Shivalingappa P., Van Westphal C., Hole R., Bodas M. (2018). Cigarette smoke-induced autophagy impairment accelerates lung aging, COPD-emphysema exacerbations and pathogenesis. *American Journal of Physiology-Cell Physiology*.

[44] Peters C., Kabelitz D., Wesch D. (2018). Regulatory functions of *γδ* T cells. *Cellular and Molecular Life Sciences*.

[45] Szabo M., Sárosi V., Balikó Z. (2017). Deficiency of innate-like T lymphocytes in chronic obstructive pulmonary disease. *Respiratory Research*.

[46] Cheng M., Hu S. (2017). Lung-resident *γδ*T cells and their roles in lung diseases. *Immunology*.

[47] Born W. K. (2017). Gammadelta T cells and B cells. *Advances in Immunology*.

[48] Witherden D. A., Johnson M. D., Havran W. L. (2018). Coreceptors and their ligands in epithelial gammadelta T cell biology. *Frontiers in Immunology*.

[49] Baral P., Umans B. D., Li L. (2018). Nociceptor sensory neurons suppress neutrophil and *γδ* T cell responses in bacterial lung infections and lethal pneumonia. *Nature Medicine*.

[50] Shyamsundar M., McAuley D. F., Ingram R. J. (2014). Keratinocyte growth factor promotes epithelial survival and resolution in a human model of lung injury. *American Journal of Respiratory and Critical Care Medicine*.

[51] Xu S. C., Kuang J. Y., Liu J., Ma C. L., Feng Y. L., Su Z. G. (2012). Association between fibroblast growth factor 7 and the risk of chronic obstructive pulmonary disease. *Acta Pharmacologica Sinica*.

[52] Lu Y., Liu F. (2018). Treatment of inflammation with Chinese medicine. *Asia-Pacific Traditional Medicine*.

[53] Ma C., Geng Y. (2006). Discussion on the application of Chinese medicine to regulate and induce systemic inflammatory response syndrome and compensatory anti-inflammatory response syndrome. *Chinese Journal of Integrated Traditional and Western Medicine in Intensive and Critical Care*.

[54] Savale L., Tu L., Rideau D. (2009). Impact of interleukin-6 on hypoxia-induced pulmonary hypertension and lung inflammation in mice. *Respiratory Research*.

[55] Tabata C., Tabata R., Takahashi Y., Nakamura K., Nakano T. (2015). Thalidomide prevents cigarette smoke extract-induced lung damage in mice. *International Immunopharmacology*.

[56] Gorska K., Nejman-Gryz P., Paplinska-Goryca M., Korczynski P., Prochorec-Sobieszek M., Krenke R. (2018). Comparative study of IL-33 and IL-6 levels in different respiratory samples in mild-to-moderate asthma and COPD. *COPD: Journal of Chronic Obstructive Pulmonary Disease*.

[57] Hoshino T., Kato S., Oka N. (2007). Pulmonary inflammation and emphysema. *American Journal of Respiratory and Critical Care Medicine*.

[58] Lee J. S., Rosengart M. R., Kondragunta V. (2007). Inverse association of plasma IL-13 and inflammatory chemokines with lung function impairment in stable COPD: a cross-sectional cohort study. *Respiratory Research*.

